# Is there a relationship between attitudes of general practitioners/family doctors and attitudes of their patients regarding industry-sponsored clinical investigations? A cross-sectional survey in a convenience sample of doctors and patients across nine European countries

**DOI:** 10.3325/cmj.2024.65.327

**Published:** 2024-08

**Authors:** Maja Marković Zoya, Ksenija Kranjčević, Jasna Vučak, Ljubin Sukriev, Josep Vidal-Alaball, Catarina Matos de Oliveira, Donata Kurpas, İlhami Ünlüoğlu, Zaim Jatić, Nevena Todorović, Darinka Punoševac, Marta Tundzeva, Milena Cojić, M Mümtaz Maziociğlu, Vladimir Trkulja

**Affiliations:** 1 ICON d.o.o., Zagreb, Croatia; 2 Department of Family Medicine, Zagreb University School of Medicine, Zagreb, Croatia; 3 Department of Family Medicine, Rijeka University School of Medicine, Rijeka, Croatia; 4 Association of General Practice/Family Medicine South East Europe; 5 Central Catalonia Research Support Unit, Jordi Gol i Gurina Foundation, Gerència Atenció Primària i a la Comunitat Catalunya Central, Catalan Health Institute, Sant Fruitós de Bages, Spain; 6 Center for Health Technology and Services Research (CINTESIS), Faculty of Medicine of the University of Porto, Porto, Portugal; 7 Family Medicine Department, Health Sciences Faculty, Wroclaw, Poland; 8 Department of Family Medicine, Eskisehir Osmangazi University (ESOGU), Eskisehir, Turkey; 9 Department of Family Medicine, Faculty of Medicine, University of Sarajevo, Sarajevo, Bosnia and Herzegovina; 10 Department of Family Medicine, Faculty of Medicine, University of Banja Luka, Banja Luka, Bosnia and Herzegovina; 11 Section of General Medicine, Serbian Medical Society, Belgrade, Serbia; 12 Center for Family Medicine, University of St. Cyril and Methodius, Skopje, Republic of North Macedonia; 13 Department of Family Medicine, University of Montenegro, Podgorica, Montenegro; 14 Department of Family Medicine, Ericyes Faculty of Medicine University of Kayseri, Kayseri, Turkey; 15 Department of Pharmacology, Zagreb University School of Medicine, Zagreb, Croatia

## Abstract

**Aim:**

To assess the relationship between the attitudes of general practitioners/family medicine doctors (GP/FD) and of their patients toward industry-sponsored clinical research.

**Methods:**

A cross-sectional survey included volunteer GPs/FDs who then enrolled and interviewed their patients. Data were analyzed in hierarchical models (patients nested in GPs/FDs, nested in countries/regions).

**Results:**

A total of 201 GPs/FDs from nine European countries responded to the invitation and enrolled 995 of their patients. We observed mild associations between some of the GPs/FDs’ attitudes (general opinion on sponsored clinical studies, appreciation of the general values of such studies, views about the importance of participant protection/privacy) and some of the patients’ attitudes (appreciation of the general values and of risks associated with sponsored clinical studies, importance assigned to potential personal benefits from participation). We observed no association between GPs/FDs’ attitudes and patients’ willingness to participate in such studies. However, willingness to participate increased with higher patients’ appreciation of the general values of sponsored studies, decreased with higher patients’ appreciation of associated risks, and showed a quadratic trend across the levels of importance assigned by patients to potential personal benefits (willingness was higher when the assigned importance was very low or very high). More importance to GP/FD’s advice in this respect was assigned by patients who assigned more importance to potential personal benefits, who were better educated, and who resided in rural/suburban dwellings.

**Conclusions:**

In the present convenience sample, lay-person attitudes about and willingness to participate in industry-sponsored clinical studies were associated with the attitudes of their GPs/FDs.

The development of medicinal products and medical devices intended for the diagnosis, prevention, and treatment of human diseases is highly regulated, and typically includes explicitly proving their clinical efficacy and safety before they could be approved for human use (1,2). Clinical investigations conducted for this purpose are sponsored by the companies developing such products. However, although conducted for a pragmatic purpose of developing a commercial product, industry-sponsored clinical investigations have broader beneficial impacts: i) the process of assessment of a specific product that might improve medical practice commonly also contributes to other aspects of general medical knowledge (3); ii) participants may experience benefits of new and improved procedures/treatments that are otherwise unavailable (3); iii) hosting clinical research positively affects the development of infrastructure and medical expertise of participating investigators and institutions, with eventually beneficial impact on health service outcomes (4,5). Setting-up a clinical investigation requires that a number of scientific, methodological, legislative, and ethical facets are considered (6). Those pertaining to intended commercial products are particularly strictly regulated, and their proper execution requires the engagement of companies providing specialized services. As a consequence, an entire “clinical trials industry” has emerged, and hosting industry-sponsored clinical investigations has beneficial local economic impacts (7).

Analyses of databases that keep information about clinical research (eg, ClinicalTrials.gov, the International Clinical Trials Registry Platform by World Health Organization) demonstrate that most of the industry-sponsored (and other) clinical research is conducted in high- and upper-middle-income countries (8,9). Moreover, some sociodemographic groups (even in countries with a high “intensity” of clinical research) are constantly underrepresented as participants in clinical investigations (10,11). These facts generate several issues (10-14): i) the concept of equity/fairness in opportunities to benefit individuals and communities is disturbed; ii) participation in clinical research builds trust in medical knowledge and institutions; hence, lack of opportunities to do so may result in distrust in medical science/profession, which is an important obstacle to delivering and receiving effective medical care; iii) if clinical research participants do not adequately (geographical, demographic, genetic, socio-economic particulars) represent the target population, generalizability of observations is limited; iv) clinical studies (industry or non-industry sponsored) commonly fail to recruit study participants. While the above problems are complex (10,12) and require comprehensive approaches (11,13), at the level of an individual potential research participant, the common reasons include the following: i) limited awareness about existence of clinical research and limited information/sources of information about particular research; ii) limited willingness to participate, typically “driven” by skepticism about sponsors’ motivation and objectives, associated benefits and risks, and general lack of knowledge about ethical and legal aspects (14-17). Higher patients’ awareness about the general benefits of clinical research, and about potential individual benefits that may arise from participation in a clinical investigation, as well as the awareness about participant protection measures and ethical aspects, all positively correlate with a higher willingness to participate in clinical research (15).

Considering their role of “first-line” contacts with a health care system, primary care providers, eg, in Europe typically general practitioners/family medicine doctors (GPs/FDs), might be a source of relevant and accurate information in this respect: they refer their interested patients to specific clinical research evaluating new therapies, provide information about developing treatments and results of medical research, and prime them for potential future clinical research participation (18). The present investigation addressed this last aspect of the potential role of GPs/FDs in the context of clinical studies. We aimed to assess, in a convenience sample of European GPs/FDs and their patients, whether there was a relationship between GPs/FDs’ attitudes toward industry-sponsored clinical investigations and their patients’ attitudes and willingness to participate in these trials. We targeted no specific patient group, but rather a haphazard sample of consenting participants who, during the survey period, visited their GP/FD for any reason. The hypothesis that the two could be associated was based on the rationale that some of information or views might have been “transferred” either during previous doctor-patient contact or during the brief but structured interview conducted for the present purpose.

## Participants and methods

### Study outline

Croatian Association of Teachers in General Practice/Family Medicine (DNOOM) intended to evaluate the attitudes and knowledge of Croatian GPs/FDs about the concept of evidence based medicine (EBM), (sponsored) clinical investigations, and views and practical obstacles in the implementation of EBM principles in their daily practice. As an extension of this endeavor, we conceived a cross-sectional survey planned to include, on a voluntary basis, GPs/FDs and their patients from Croatia and other European countries. The intention of the study was to assess the relationship between the GPs/FDs’ and patients’ attitudes toward industry-sponsored clinical research. This intention was presented at the annual Croatian Congress of Family Medicine, and all registered GPs/FDs in Croatia were invited to participate, directly via e-mails on three occasions during 2019. The survey was also presented at the 2019 Conference of the Association of General Practice/Family Medicine of South-East Europe and at the annual meeting of the European General Practice Research Network. The invitation to participate was extended to national societies. Societies from eight countries responded to the invitation: Bosnia and Herzegovina, Serbia, Montenegro, the Republic of Northern Macedonia, Poland, Turkey, Portugal, and Spain. Presidents of the responding societies were appointed national co-ordinators, and through multilateral consultations it was agreed that a minimum of 20 GPs/FDs from each country could be enrolled into the survey by June 1, 2020. National study co-ordinators extended direct invitations via e-mails to registered GPs/FDs, and repeated the invitations until this minimum targeted number of participants was recruited. The survey included two parts: one was intended for GPs/FDs and the other one for their patients. To minimize the burden for the GPs/FDs (and thus preserve the feasibility of the survey), each included GP/FD was to enroll five consenting patients: on each day of the week, consecutive visiting patients were to be invited, and the first consenting patient was to be enrolled. Each participating GP/FD received a unique code, and their identity was known only to the national co-ordinators. Each included patient also had a unique code, and their identity was known only to their GP/FD. The survey questionnaires and instructions explaining its purpose, anonymity, and ethical considerations, all in local languages, were delivered to the participating GPs/FDs electronically. The questionnaires were filled-out electronically. First, GPs/FDs filled-out their part of the survey. The patients’ part was conducted in the form of an interview: GPs/FDs interviewed the patients, offered additional explanations/consultations when needed, and entered their replies, as well as the interview date into the electronic form. Upon completion, the electronic forms were coded and locked.

The Croatian part of the survey was approved by the Ethics Committee of University of Split, School of Medicine. National co-ordinators were responsible for obtaining ethical approvals in line with the respective national legislation. Ethical approvals were obtained in all participating countries.

### Instruments

GP/FD questionnaire aimed to capture: 1. general information (age, sex, years of experience as a GP/FD, training specialization in general practice/family medicine, participation in education of students/residents); 2. general information on their practices (rural/suburban/urban community, the number of registered patients, estimated average number of patient contacts per day); 3. personal experience with sponsored clinical research as investigators (yes/no) or participants (eg, healthy volunteers; yes/no); 4. personal views on several domains (all scored on five-level numerical rating scales): i) general attitude toward industry-sponsored clinical research (1 – negative to 5 – positive); ii) importance assigned to participant protection measures in sponsored research, based on two items (1 – disagree to 5 – agree): (a) strict confidentiality and protection of participant privacy in sponsored research is very important, (b) sponsors’ accountability for damage is very important; iii) assigned level of importance of sponsored research for practical medicine, based on four items (1 – disagree to 5 – agree): (a) sponsored clinical research generates important evidence relevant for rational medical practice, (b) principles of evidence-based medicine should be promoted, (c) evidence generated in sponsored clinical research reflects on my daily practice, (d) implementation of evidence-based procedures improves patient care; iv) skepticism about implementation of evidence-based principles – two items (1 – disagree to 5 – agree) (a) evidence generated in sponsored clinical research is of a limited value for general practice/family medicine; (b) adopting evidence-based procedures/principles of evidence-based medicine is a valuable intention, but its implementation poses an extra load to the already overloaded GPs/FDs.

Patient questionnaire aimed to capture: 1. general information (age, sex, and education, categorized as “low” [elementary schooling or less], “medium” [high-school graduates or equivalent], or “higher” [college/university graduates or higher]); 2. main health issues (if any – targeted questions by organ systems); 3. willingness to participate in potential industry-sponsored clinical research (yes/no/undecided); 4. importance assigned to GP/FD consultation in this respect (1 – least important to 5 – most important); 5. agreement about the general values of sponsored clinical research based on two items (1 – disagree to 5 – agree): (a) industry-sponsored clinical research contributes to improvement of population health and development, (b) increasing the number of sponsored clinical research would contribute to improvement of people’s health in your country; 6. concerns about risks related to participation in clinical research based on two items (1 – disagree to 5 – agree): (a) industry-sponsored clinical research is unethical, (b) participation in an industry-sponsored clinical investigation could harm my health; 7. importance assigned to potential individual benefits related to participation in industry-sponsored clinical research, based on 3 items (1 – least important to 5 – most important): (a) by participating in a sponsored clinical trial, I could get new treatments that are otherwise unavailable, (b) participation in a sponsored clinical trial could allow me to continue using new treatments even after the trial completion, (c) the sponsor is liable for any harm that I could experience in a sponsored clinical investigation.

The Croatian version of the questionnaires was evaluated in a pilot study. Of the 20 randomly selected Croatian GPs/FDs, members of DNOOM, 15 responded and, together with the investigators, formed a focus group that discussed whether the questionnaires were understandable to GPs/FDs and whether they were likely to capture the intended constructs. They each enrolled 5 of their patients (total N = 75) and reported back on the time needed to conduct the patient interview and fill-out the patient’s questionnaire, and on the level of understanding of questions directed to patients. Patients’ answers to 7 items targeting attitudes were subjected to exploratory factor analysis (EFA). Three factors were identified (Supplemental Material, Section A(Supplementary Material)) corresponding to three intended latent constructs, and were named as follows: (i) agreement about the general values of industry-sponsored clinical research (two items), (ii) agreement about potential risks (two items), and (iii) importance assigned to potential personal benefits (three items). In collaboration with national co-ordinators in countries with languages closely similar to Croatian (Bosnia and Herzegovina, Serbia, Montenegro), the Croatian version was adapted for the local purposes. The Croatian version was also translated and backtranslated to/from the English language. A suitable English version was then conveyed to other national co-ordinators (Northern Macedonia, Poland, Turkey, Spain, Portugal), who were responsible to translate/backtranslate the questionnaire to the local language.

### Outcomes and predictors of primary interest

Outcomes of interest were as follows: a) the (anticipated) constructs illustrating patients’ attitudes about industry-sponsored clinical research that were to be confirmed by EFA: (i) agreement about the general values of such research, (ii) agreement about potential risks, and (iii) importance assigned to potential personal benefits. Predictors of primary interest were those illustrating GPs/FDs’ attitudes. Other patient-level (eg, demographics, education) and GP/FD-level characteristics were considered as covariates; b) patients’ general willingness to participate in a sponsored clinical investigation. Predictors of primary interest were GPs/FDs’ and patients’ attitudes (other characteristics = covariates); and c) importance assigned to advice from a GP/FD in this respect.

### Missing data/data imputation

GPs/FDs were explicitly asked to carefully fill out the parts of questionnaires pertaining to “general information” about themselves and about their practices, and general patient information (demographics, education, major health issues). We expected that some of the items addressing attitudes (ie, some of the predictors of primary interest and some of the outcomes) would occasionally remain unanswered either by chance, error, or respondents’ indecisiveness. Regarding GP/FD attitude-related items, there were 1%-3% missing data (Supplemental Material, Section B(Supplementary Material)), while regarding patients’ attitude-related items there were 4%-6% missing data (Supplemental Material, Section C(Supplementary Material)). We used multiple imputation to impute missing data (Supplemental Material, Section B, Section C(Supplementary Material)).

### Forming attitude-related predictors and outcomes

Items related to GPs/FDs’ attitudes (n = 9) were subjected to EFA, and 4 predictors were identified: (i) general attitude toward industry-sponsored clinical research; (ii) agreement about the general values of sponsored clinical research (4 items); (iii) importance assigned to participant protection and privacy in sponsored studies (2 items); (iv) skepticism about implementation of evidence-based principles in general practice/family medicine (2 items) (Supplemental Material, Section D(Supplementary Material)). General attitude toward sponsored clinical research was considered a 5-level ordinal variable. For other predictors, numerical values were simple additive combinations of individual item values (range 4 to 20 or 2 to 10) and were considered as ordered categories (higher values indicating higher agreement/importance).

Items related to patients’ attitudes (n = 7) were also subjected to EFA, and the same three outcomes were identified as in the pilot data: (i) agreement about general values of industry-sponsored clinical research (two items), (ii) agreement about potential risks (two items), and (iii) importance assigned to potential personal benefits (three items) (Supplemental Material, Section E(Supplementary Material)). Numerical values were simple additive combinations of individual items (range 2-10, or 3-15, indicating higher levels of agreement/importance), and were treated as ordered categories.

### Data analysis

Outcomes were analyzed by fitting generalized linear three-level hierarchical models: patients (first level) were nested in GPs/FDs (second level), who were nested in countries organized in three regions (third level): i) Croatia – a small country, the “youngest” European Union member state, with a low number of ongoing clinical trials registered in the European Union Drug Regulating Authorities Clinical Trials Database (EudraCT) compared with similarly sized Central Eastern-European EU member states (14); ii) Serbia, Bosnia and Herzegovina, Montenegro, and the Republic of North Macedonia – Croatia’s neighboring countries, roughly similar in size, with a similar concept of primary health care, and with similarly low presence of sponsored clinical studies; iii) Portugal, Spain, Poland (“old” EU member states) each with a large number of industry-sponsored clinical investigations conducted every year (19), and Turkey, a large country with constantly increasing number of industry-sponsored trials, and with a seemingly strong nationwide incentive to further increase the number (20) (Supplemental Material, Section F(Supplementary Material)). Patient attitude-related outcomes (agreement about general values of industry-sponsored clinical research, agreement about potential risks, and importance assigned to potential personal benefits), willingness to participate, and importance assigned to GP/FD advice in this respect were ordered categories or binary outcomes and were analyzed in cumulative or binary logit models. The need to fit hierarchical models was imposed by the data structure: to estimate associations between second-level predictors (GPs/FDs’ views/attitudes) and first-level outcomes (patients’ views/attitudes), correlations between outcomes “within” the same GP/FD and “within” the same region had to be accounted for. Estimates were generated with adjustments for the first-level (patient) and second-level (GPs/FDs) covariates. To indicate the fractions (%) of total variability attributable to variability across GPs/FDs within regions, and across regions, for each outcome we calculated intraclass correlation coefficients (ICC). We implemented maximum likelihood estimation with Laplace approximation in SAS for Windows 9.4 (SAS Inc. Cary, NC, USA) (21).

## Results

### Participants

**Figure 1:**
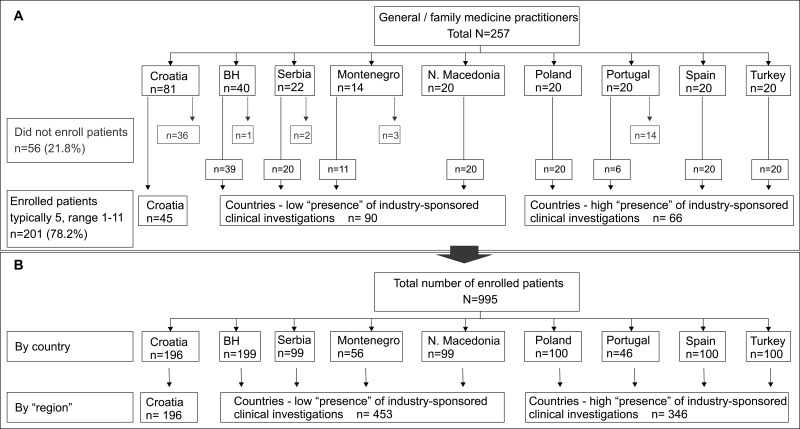
Structure of the general practitioners/family doctors (GPs/FDs) and their patients who participated in the present survey. **A.** General practitioners/family doctors – overall, by country, and by region defined based on the presence of industry-sponsored clinical investigations (see Data analysis and Supplemental Material, Section F(Supplementary Material)). Gray lines/boxes/font depict GPs/FDs who returned questionnaires but enrolled no patients and were hence not included in the present analysis. **B.** Patients – overall, by country and by region. BH – Bosnia and Herzegovina

A total of 257 GPs/FDs returned the questionnaires (Figure 1), 56 (21.8%) of whom enrolled no patients: 201 GPs/FDs and 995 of their patients were included in the present analysis (Figure 1). The participating GPs/FDs were predominantly women, on average 40-50 years of age (Table 1), practicing in urban dwellings, who were specialists in family medicine, commonly involved in the education of students/residents, 50% of whom had had experience as investigators in clinical research (Table 1). They typically managed around 2000 registered patients, with an average of around 50 patient contacts per day (Table 1). Most GPs/FDs expressed a negative general attitude toward industry-sponsored clinical research: on a scale from 1 – negative to 5 – positive, only 25%-35% scored 3 or higher (Table 1). Most of them generally assigned low importance to participant protection in sponsored studies: on a scale from 2 – low importance to 10 – high importance, the median score was 2 (Table 1). On the other hand, they consistently expressed a high agreement about general values of such research (median 19 with maximum possible score of 20) (Table 1), and expressed (only) mild skepticism about applicability of the results of such studies in their daily practice: on a scale from 2 –low skepticism to 10 – high skepticism, the median score was 5 (Table 1). In agreement, 40%-45% of them estimated that 80% or more of their daily practice was evidence-based (Table 1). The 56 not included GPs/FDs displayed similar characteristics (Supplemental Material, Section G(Supplementary Material)).

**Table 1:** Characteristics of general practitioners/family medicine doctors (GPs/FDs) included in the analysis (GPs/FDs who recruited at least one patient) – overall and by region*

	All	Croatia	BH, Serb, MN, North Maced.	Portugal, Spain, Poland, Turkey
N	201	45	90	66
Female	154 (76.6)	39 (86.7)	77 (85.6)	38 (57.6)
Age	46 ± 11 (25-69)	49 ± 9 (26-65)	48 ± 9 (25-64)	40 ± 12 (26-69)
Years of experience as GP/FD	16 ± 10 (1-40)	21 ± 9 (1-39)	18 ± 9 (1-35)	12 ± 10 (1-40)
Dwelling				
urban	136 (67.7)	37 (82.2)	67 (74.4)	32 (48.5)
suburban/rural	65 (32.3)	8 (17.8)	23 (25.6)	34 (51.5)
Specialist	164 (81.6)	38 (84.4)	81 (90.0)	45 (68.2)
Educates students/resident	144 (71.6)	36 (80.0)	63 (70.0)	45 (68.2)
Has been an investigator in a clinical study	101 (50.2)	21 (46.7)	48 (53.3)	32 (48.5)
Has been a participant in a clinical study	37 (18.4)	5 (11.1)	19 (21.1)	13 (19.7)
Registered patients ( × 1000)	1.92 (1.51-2.10)	1.72 (1.50-1.96)	1.88 (1.70-2.04)	2.0 (1.29-2.92)
Average daily patient contacts	45 (35-60)	80 (65-98)	45 (40-50)	30 (25-45)
Attitude toward sponsored studies				
1 – negative	86 (42.8)	15 (33.3)	38 (42.2)	33 (50.0)
2	49 (24.4)	16 (35.6)	16 (17.8)	17 (25.8)
3	28 (13.9)	6 (13.3)	15 (16.7)	7 (10.6)
4	29 (14.4)	8 (17.8)	15 (16.7)	6 (9.1)
5 – positive	9 (4.5)	0	6 (6.7)	3 (4.5)
Agreement about general values of industry-sponsored research^†‡^	19 (17-20)	19 (17-20)	19 (17-20)	18 (16-19)
Importance assigned to subject protection/privacy^†§^	2 (2-3)	2 (2-3)	2 (2-3)	2 (2-4)
Skeptical about implementation of evidence-based procedures^†§^	5 (3-7)	5 (2.5-6)	4 (2-6)	6 (4-8)
Own practice is evidence-based?				
<5%	8 (4.0)	0	7 (7.8)	1 (1.5)
around 10%-30%	6 (3.0)	0	3 (3.3)	3 (4.5)
around 40%-50%	20 (9.9)	4 (8.9)	7 (7.8)	9 (13.6)
around 60%-70%	72 (35.8)	20 (44.5)	30 (33.3)	22 (33.3)
around 80%	58 (28.9)	15 (33.3)	26 (28.9)	17 (25.8)
90%-100%	31 (15.4)	6 (13.3)	13 (14.4)	12 (18.2)
did not answer	6 (3.0)	0	4 (4.4)	2 (3.0)

*Bosnia and Hercegovina [BH], Serbia [Serb], Montenegro [MN] and North Macedonia were considered countries with a “lower presence” of industry-sponsored clinical investigations, whereas Portugal, Spain, Poland, and Turkey were considered countries with a “higher presence” of such studies) (see Data Analysis and Supplemental Material, Section F(Supplementary Material))˝. Data are mean ± standard deviation (range), median (quartiles) or count (percent).

†Variables identified in exploratory factor analysis. Values are sums of individual items.

‡Minimum possible score 4, maximum 20, mid-point 12.

§Minimum possible score 2, maximum 10, mid-point 6. Higher values = higher level or agreement/assigned importance/skepticism.

The enrolled patients were on average 52 years old, with a slight predominance of women (Table 2), most suffering from some chronic condition; however around 27% were generally healthy (Table 2). At least 50% stated that, if offered, they would participate in an industry-sponsored clinical investigation (Table 2), around 25% explicitly declined such a possibility, while around 20% were undecided (Table 2). The majority (60%-70%) assigned little importance to a possible consultation with their GP/FD in this respect (scored 1 or 2 on a 1 to 5 scale) (Table 2). Around 50% of the patients expressed high agreement about the general values of sponsored clinical studies (scored 9-10 on a 2 to 10 scale) (Table 2), and somewhat more than 50% expressed low agreement with the claims indicating health risks associated with such studies (scores 2-4 or 5-6 on a 2-10 scale) (Table 2). Most patients (60%-70%) assigned low importance (scores 3-4 or 5-6 on a 3- 15 scale) to potential personal benefits that they might experience from participation in industry-sponsored clinical studies (Table 2).

**Table 2:** Patients’ characteristics, overall and by region

	All	Croatia	BH, Serb, MN, North Maced.	Portugal, Spain, Poland, Turkey
N	995	196	453	346
Female	583 (59.0)	127 (64.8)	270 (60.4)	186 (53.8)
Age	52 ± 16 (18-91)	52 ± 15 (19-82)	52 ± 15 (20-86)	50 ± 17 (18-91)
Dwelling				
urban	651 (65.4)	154 (78.6)	335 (73.9)	162 (46.8)
suburban/rural	344 (34.6)	42 (21.4)	118 (26.1)	184 (53.2)
Education				
less than high-school	50 (5.0)	9 (4.6)	37 (8.2)	4 (1.2)
high school or equivalent	544 (54.7)	108 (55.1)	233 (51.4)	203 (58.7)
higher education (≥college)	401 (40.3)	79 (40.3)	183 (40.4)	139 (40.2)
Major current health issues				
generally healthy	268 (26.9)	56 (28.6)	83 (18.3)	129 (37.3)
diabetes mellitus	156 (15.7)	15 (7.7)	86 (19.0)	55 (15.9)
gastrointestinal tract disorders	148 (14.9)	29 (14.8)	71 (15.7)	48 (13.9)
musculoskeletal/connective tissue disorders	138 (13.9)	27 (13.8)	74 (16.3)	37 (10.7)
respiratory tract disorders	129 (13.0)	26 (13.3)	52 (11.5)	51 (14.7)
coronary or cerebrovascular disease	121 (12.2)	22 (11.2)	57 (12.6)	42 (12.1)
neurological disorders	81 (8.1)	16 (8.2)	47 (10.4)	18 (5.2)
renal and urinary tract disorders	79 (7.9)	17 (8.7)	38 (8.4)	24 (6.9)
mental disorders	57 (5.7)	11 (5.6)	29 (6.4)	17 (4.9)
malignancy	34 (3.4)	15 (7.7)	12 (2.7)	7 (2.0)
If offered, would participate in an industry-sponsored study?				
yes	557 (56.0)	120 (61.2)	266 (58.7)	171 (49.4)
no	236 (23.7)	48 (24.5)	95 (21.0)	93 (26.9)
undecided	202 (20.3)	28 (14.3)	92 (20.3)	82 (23.7)
In this respect, the advice from my GP is				
1 (least important)	646 (64.9)	138 (70.4)	296 (65.3)	212 (61.3)
2	139 (14.0)	45 (23.0)	32 (7.1)	62 (17.9)
3	89 (8.9)	8 (4.1)	47 (10.4)	34 (9.8)
4	57 (5.7)	3 (1.5)	35 (7.7)	19 (5.5)
5 (most important)	64 (6.4)	2 (1.0)	43 (9.5)	19 (5.5)
Agreement about general values of sponsored studies^†^ (total item scores)				
2-4 (lowest)	48 (4.8)	5 (2.5)	22 (4.9)	21 (6.1)
5-6	174 (17.5)	38 (19.4)	70 (15.4)	66 (19.1)
7-8	256 (25.7)	47 (24.0)	115 (25.4)	94 (27.2)
9-10 (highest)	517 (52.0)	106 (54.1)	246 (54.3)	165 (47.7)
Agreement about potential risks of sponsored studies^†^ (total item scores)				
2-4 (lowest)	231 (23.2)	46 (23.5)	123 (27.1)	62(17.9)
5-6	352 (35.4)	74 (37.8)	152 (33.5)	126 (36.4)
7-8	296 (29.7)	55 (28.1)	118 (26.1)	123 (33.6)
9-10 (highest)	116 (11.7)	21 (10.7)	60 (13.3)	35 (10.1)
Importance assigned to potential personal benefits^†^ (total item scores)				
3-4 (lowest)	417 (41.9)	114 (58.2)	196 (43.3)	107 (30.9)
5-6	220 (22.1)	40 (20.4)	81 (17.9)	99 (28.6)
7-8	140 (14.1)	23 (11.7)	53 (11.7)	64 (18.5)
9-10	100 (10.1)	13 (6.6)	40 (8.8)	47 (13.6)
11-12	41 (4.1)	3 (1,5)	24 (5.3)	14 (4.1)
13-15 (highest)	77 (7.7)	3 (1.5)	59 (13.0)	15 (4.3)

*Data are mean ± standard deviation (range) or count (percent).

†Variables identified in exploratory factor analysis. Values are sums of individual items: higher scores = higher level of agreement/assigned importance.

### Relationship between GPs/FDs’ and patients’ attitudes toward industry-sponsored clinical research

For all three outcomes (patients’ attitudes): agreement about the general values of sponsored studies, agreement about potential risks and importance assigned to potential personal benefits, the variability across regions was none or minimal (ICC = 0.0, 0.01%, and 4.0%, respectively, see footnote to Table 3), and variability across GPs/FDs within regions was moderate to considerable (ICC = 14.7%, 15.8%, and 36.1%, respectively). More positive general GPs/FDs’ attitude (OR = 0.70, 95%CI 0.50-0.98) and higher importance assigned to participant protection (OR = 0.73, 0.52-0.99) were univariately associated with lower odds of higher patients’ agreement about general values (Table 3). Of the covariates, older GPs/FDs’ age was associated with higher odds (OR = 1.14, 1.05-1.24), older patients’ age was associated with lower odds (OR = 0.95, 0.91-0.99), and higher education was associated with higher odds (OR = 1.90, 1.30-2.78) of higher patients’ agreement about general values (Table 3). With adjustment for all covariates, the association of the GPs/FDs’ importance assigned to participant protection and this outcome was reduced (OR = 0.76, 0.52-1.03) (Table 3), while that of the GPs/FDs’ general attitude remained unchanged (Table 3). Higher GPs/FDs’ agreement about general values was associated with lower odds of higher patient agreement about potential risks (univariate OR = 0.69, 0.47-1.02, with adjustment for all covariates OR = 0.65, 0.43-0.97) (Table 3). No covariate was clearly associated with this outcome (Table 3). More positive GPs/FDs general attitude was univariately associated with higher odds of higher importance assigned by patients to potential personal benefits (OR = 3.35, 2.08-5.39) (Table 3), while higher GPs/FDs agreement about general values was associated with lower odds (OR = 0.47, 0.27-0.83) (Table 3). Of the covariates, GPs/FDs educator status (OR = 0.49, 0.29-0.81) and experience of an investigator in sponsored studies (OR = 0.58, 0.36-0.91), as well as higher patients’ education (OR = 0.67, 0.45-0.99), were associated with lower odds of higher patient-assigned importance to personal benefits (Table 3), whereas generally healthy patients were more likely to assign higher importance to potential personal benefits (OR = 2.25, 1.60-0.99). With adjustment for all covariates, the associations between GPs/FDs’ attitudes and the outcome were not substantially changed (Table 3) (Supplemental Material, Section H(Supplementary Material)).

**Table 3:** The relationship between general practitioners/family doctors’ (GPs/FDs’) (predictors of primary interest) and patients’ attitudes about industry-sponsored clinical investigations (outcomes). Modeled is the probability of higher-ordered level: odds ratios (OR)>1.0 indicate positive association, ORs <1.0 indicate negative association. Outcomes were ordered categories with four or six levels (see Table 2).

	Outcomes – patients’ attitudes		
Predictors: GPs/FDs’ attitudes	agreement about general values*	agreement about potential risks^†^	importance of personal benefits^‡^
Univariate –each predictor separately			
General attitude (3-5 vs 1-2)	0.70 (0.50-0.98)	1.16 (0.82-1.65)	3.35 (2.08-5.39)
Importance of subject protection (4-10 vs 2-3)	0.73 (0.52-0.99)	1.05 (0.72-1.55)	1.12 (0.64-1.98)
Agreement about general values (17-20 vs <17)	1.01 (0.68-1.51)	0.69 (0.47-1.02)	0.47 (0.27-0.83)
Skepticism about implementation			
intermediate (5-7) vs low (2-4)	0.83 (0.64-1.29)	1.04 (0.68-1.60)	1.12 (0.66-1.92)
high (8-10) vs low (2-4)	0.80 (0.52-1.22)	1.19 (0.83-1.71)	0.80 (0.42-1.51)
Multivariate model – only covariates			
GPs/FDs’ characteristics			
age (by 5 years)	1.14 (1.05-1.24)	0.96 (0.88-1.04)	1.04 (0.92-1.17)
male sex	0.98 (0.66-1.45)	1.23 (0.84-1.81)	0.77 (0.44-1.32)
urban dwelling	1.22 (0.83-1.77)	0.89 (0.62-1.29)	0.76 (0.46-1.26)
family medicine specialist	0.75 (0.47-1.16)	0.78 (0.50-1.19)	1.43 (0.79-2.58)
educates students/residents	0.87 (0.59-1.27)	0.95 (0.65-1.38)	0.49 (0.29-0.81)
has been an investigator	1.03 (0.73-1.44)	0.77 (0.55-1.07)	0.58 (0.36-0.91)
practice ≥80% evidence-based	1.18 (0.85-1.66)	1.06 (0.77-1.48)	1.31 (0.83-2-05)
Patient characteristics			
age (by 5 years)	0.95 (0.91-0.99)	1.03 (0.98-1.07)	1.02 (0.97-1.06)
male sex	1.01 (0.78-1.31)	0.93 (0.72-1.19)	1.02 (0.78-1.33)
generally healthy	0.95 (0.69-1.31)	0.88 (0.65-1.20)	2.25 (1.60-3.16)
education			
high-school vs less	1.33 (0.72-2.46)	1.57 (0.87-2.83)	0.92 (0.48-1.74)
college or higher vs less	1.90 (1.30-2.78)	0.97 (0.68-1.39)	0.67 (0.45-0.99)
Each predictor separately + covariates			
General attitude	0.67 (0.47-0.97)	1.20 (0.83-1.73)	3.35 (2.05-5.49)
Importance of subject protection	0.76 (0.52-1.03)	1.06 (0.72-1.55)	0.98 (0.58-1.66)
Agreement about general values	1.05 (0.71-1.57)	0.65 (0.43-0.97)	0.43 (0.25-0.75)
Skepticism about implementation			
intermediate vs low	0.75 (0.55-1.19)	1.04 (0.71-1.50)	1.01 (0.60-1.70)
high vs low	0.76 (0.49-1.18)	0.91 (0.59-1.41)	0.80 (0.44-1.52)

*Intraclass correlation coefficient (ICC) for regions = 0.0; for GPs/FDs within regions ICC = 14.7%; variability to be accounted by patients: 85.3%.

^†^For regions, ICCs = 0.01%, for GPs/FDs (in regions) ICC = 15.8%, variability to be accounted by patients: 84.2%.

^‡^For regions, ICC = 4.0%, for GPs/FDs (in regions) ICC = 36.1%, ICC for regions: 4.0%, variability to be accounted by patients: 59.9%.

### Relationship between GPs/FDs’ attitudes and of patients’ attitudes toward sponsored clinical research and patients’ willingness to participate

**Figure 2:**
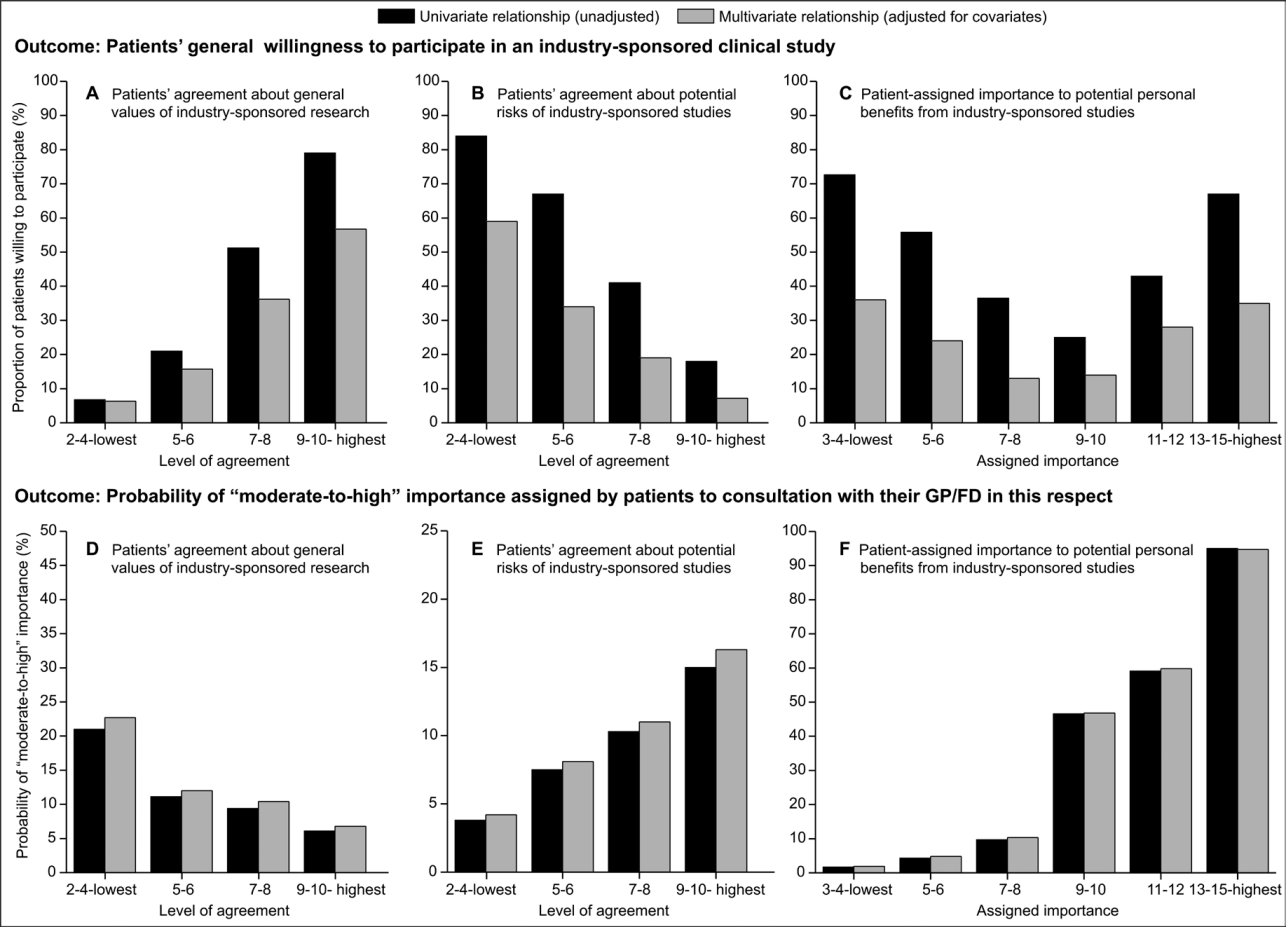
Relationship between patients’ attitudes toward industry-sponsored clinical research and (i) their (general) willingness to participate in such studies (**A-C**), and (ii) probability of assigning “moderate-to-high” importance (score 3-5 on a scale 1-lowest to 5-highest) to a consultation with or advice by their general practitioner/family doctor (GP/FD) in this respect (**D-E**). Shown are raw (unadjusted) and adjusted estimated probabilities of (i) willingness to participate (from models in Table 4) and of assigning “moderate-to-high” importance to GP/FD advice (from models in Table 5): (**A, D**) across the levels of patients’ agreement about general values of industry-sponsored studies; (**B, E**) across the levels of patients’ agreement about the risks associated with participation in such studies, and (**C, F**) across the levels of patient-assigned importance to potential personal benefits from participation in such studies.

There was practically no variability of the outcome across regions (ICC = 0.5%), and a considerable variability across GPs/FDs within regions (ICC = 24.6%) (footnote to Table 4). Higher GPs/FDs’ agreement about the general values of sponsored studies numerically was associated with higher willingness to participate, but with uncertainty (OR = 1.40, 95%CI 0.90-2.45) (Table 4), whereas all three patients’ attitude variables were associated with the outcome: willingness linearly increased with higher agreement about the general values of sponsored studies (Table 4, Figure 2A), decreased with higher agreement about potential risks (Table 4, Figure 2B), and decreased from the low level of importance that patients assigned to potential personal benefits to intermediate importance, and then again increased at the high level of importance (Table 4, Figure 2B). Of the covariates, older GPs/FDs’ age and better patients’ education were associated with higher odds of willingness to participate (Table 4), whereas older patients’ age was associated with lower odds (Table 4). With adjustment for covariates, the association between higher GPs/FDs’ agreement about the general values of sponsored studies and higher odds of willingness became clearer (OR = 1.70, 1.01-2.87), while the relationship between the patients’ attitudes and the outcome was not substantially changed (Table 4, Figure 2A-C) (Supplemental Material, Section I(Supplementary Material)).

**Table 4:** The relationship of the general practitioner/family doctors’ (GPs/FDs) and patients’ attitudes toward industry-sponsored clinical studies with patients’ willingness to participate in a hypothetical study. Modeled is the probability of “willing to participate” (vs unwilling/undecided): odds ratios (ORs)>1.0 indicate positive associations, ORs <1.0 indicate negative associations*

	OR (95% CI)
Univariate –each GPs/FDs’ attitude predictor separately	
General attitude (3-5 vs 1-2)	0.81 (0.52-1.26)
Importance of subject protection (4-10 vs 2-3)	0.66 (0.40-1.07)
Agreement about general values (17-20 vs <17)	1.48 (0.90-2.45)
Skepticism about implementation in daily practice	
intermediate (5-7) vs low (2-4)	0.88 (0.54-1.41)
high (8-10) vs low (2-4)	1.03 (0.59-1.80)
Univariate – each patients’ attitude predictor separately	
Agreement about general values of sponsored studies (4 levels)	Linear increasing trend *P* < 0.001
Agreement about potential risks of sponsored studies (4 levels)	Linear decreasing trend *P* < 0.001
Importance assigned to potential personal benefits (6 levels)	Quadratic trend *P* < 0.001
Multivariate model – only covariates	
GPs/FDs’ characteristics	
age (by 5 years)	1.26 (1.13-1.42)
male sex	0.68 (0.42-1.13)
urban dwelling	0.79 (0.49-1.28)
family medicine specialist	0.73 (0.42-1.28)
educates students/residents	1.18 (0.73-1.90)
has been an investigator	1.40 (0.92-2.14)
practice ≥80% evidence-based	1.02 (0.67-1.56)
Patient characteristics	
age (by 5 years)	0.88 (0.84-0.93)
male sex	1.04 (0.77-1.42)
generally healthy	0.70 (0.49-1.03)
education	
high-school vs less	2.54 (1.22-5.30)
college or higher vs less	2.30 (1.46-3.62)
Each GPs/FDs’ attitude predictor separately + covariates	
General attitude (3-5 vs 1-2)	0.89 (0.56-1.42)
Importance of participant protection (4-10 vs 2-3)	0.70 (0.43-1.16)
Agreement about general values (17-20 vs <17)	1.70 (1.01-2.87)
Skepticism about implementation in daily practice	
intermediate (5-7) vs low (2-4)	0.82 (0.51-1.32)
high (8-10) vs low (2-4)	1.01 (0.58-1.75)
Each patients’ attitude predictor separately + covariates	
Agreement about general values of sponsored studies (4 levels)	Linear increasing trend *P* < 0.001
Agreement about potential risks of sponsored studies (4 levels)	Linear decreasing trend *P* < 0.001
Importance assigned to potential personal benefits (6 levels)	Quadratic trend *P* < 0.001

*Intraclass correlation coefficient for regions = 0.5%; for GPs/FDs within regions ICC = 24.6%; variability to be accounted by patients: 74.9%.

### Patients’ attitudes toward industry-sponsored clinical studies and importance that they assigned to advice from their GP/FD regarding potential participation in such studies

The outcome was dichotomized as “moderate-to-high importance” (score 3-5) vs “low or very low importance” (score 1-2). There was mild variability of the outcome across regions (ICC = 7.0%), and a considerable variability across GPs/FDs within regions (ICC = 46.6%) (footnote to Table 5). In univariate analysis, probability of the outcome decreased with higher patients’ agreement about the general values of sponsored studies (Table 5, Figure 2D), and increased with higher agreement about potential risks (Table 5, Figure 2E), as well as with higher importance assigned to potential personal benefits (Table 5, Figure 2F). Patients’ willingness to participate in sponsored studies was not associated with the outcome (Table 5). Of the covariates, GPs/FDs’ male sex (OR = 0.36, 95%CI 0.15-0.84), urban dwelling (OR = 0.45, 0.21-0.94), and educator status (OR = 0.34, 0.16-0.71) were associated with lower odds of the outcome (Table 5). With adjustment for covariates, the association between the patients’ attitudes and willingness to participate was not substantially changed (Table 5, Figure 2D-F) (Supplemental Material, Section J(Supplementary Material)).

**Table 5:** Importance assigned by patients to a consultation/advice from their general practitioner/family doctor (GP/FD) regarding potential participation in an industry-sponsored clinical study. Modeled is the probability of “moderate-to-high importance” (score 3-5 on a scale from 1-lowest to 5 – highest) vs “low or very low importance” (score 1-2). Odds ratios (ORs)>1.0 indicate positive associations, ORs <1.0 indicate negative association*

	OR (95%CI)
Univariate – each patients’ attitude and willingness separately	
Agreement about general values of sponsored studies (4 levels)	Linear decreasing trend *P* < 0.001
Agreement about potential risks of sponsored studies (4 levels)	Linear increasing trend *P* < 0.001
Importance assigned to potential personal benefits (6 levels)	Linear increasing trend *P* < 0.001
Willingness to participate in a sponsored study (vs no or undecided)	0.70 (0.45-1.09)
Multivariate model – only covariates	
GPs/FDs’ characteristics	
age (by 5 years)	1.02 (0.85-1.21)
male sex	0.36 (0.15-0.84)
urban dwelling	0.45 (0.21-0.94)
family medicine specialist	1.24 (0.51-3.01)
educates students/residents	0.34 (0.16-0.71)
has been an investigator	0.70 (0.36-1.38)
practice ≥80% evidence-based	1.12 (0.57-2.21)
Patient characteristics	
age (by 5 years)	0.97 (0.91-1.04)
male sex	0.97 (0.64-1.45)
generally healthy	1.12 (0.67-1.86)
education	
high-school vs less	1.56 (0.56-4.34)
college or higher vs less	1.02 (0.54-1.92)
Each patients’ attitude and willingness separately + covariates	
Agreement about general values of sponsored studies (4 levels)	Linear decreasing trend *P* = 0.001
Agreement about potential risks of sponsored studies (4 levels)	Linear increasing trend *P* < 0.001
Importance assigned to potential personal benefits (6 levels)	Linear increasing trend *P* < 0.001
Willing to participate in a sponsored study (vs no or undecided)	0.71 (0.46-1.10)

*Intraclass correlation coefficient (ICC) for regions = 7.0%; for GPs/FDs within regions ICC = 46.6%; variability to be accounted by patients: 46.4%.

## Discussion

The present results indicate that some of the GPs/FDs’ attitudes were associated with some of the patients’ attitudes. GPs/FDs may considerably contribute to patient enrollment into clinical studies by: i) providing information to their interested patients about existing possibilities of enrollment (18,22); ii) referring their patients to a specific study (18,22); iii) providing information and advice about participation in a specific study; or iv) disseminating general information about the importance of clinical research for advancement of health care, potential benefits and risks associated with participation in clinical studies, as well as information about stringent scientific, ethical, and legal standards to which such studies must comply. Several studies in cancer patients indicated that the information and advice conveyed by primary care providers regarding patients’ decisions about trial participation were highly valued by the patients (23,24). This may help shape more affirmative attitudes of the general public about industry-sponsored clinical studies (18), and may result in higher patients’ willingness to participate in such studies, and more positive acceptance of the results of medical research (15).

The present survey intended to test if attitudes on the topic might have been “transferred” between GPs/FDs and their patients even without programmatic or educational interventions, simply as a result of their previous contacts, or (more likely) as a result of a simple, structured 15-minute interview where GPs/FDs were free to comment, provide information or guidance.

GPs/FDs’ attitudes consisted of four constructs: i) general attitude toward industry-sponsored studies; ii) appreciation of the general values of such studies; iii) importance assigned to the protection of study participants; iv) skepticism about the implementation of the results of such studies in daily practice. The patients’ attitudes were subsumed in three constructs: i) appreciation of the general values of sponsored clinical studies, ii) agreement about potential risks of such studies, iii) importance assigned to potential personal benefits of participation in such studies. Since the present sample was a convenience one, not representative for the participating countries, we did not consider possible differences between them in any aspect. It should be noted however that for all outcomes of interest the fraction of total variability that was due to variability across regions was practically non-existent (ICC = 0%-7.0%).

Patients’ appreciation of the general values of industry-sponsored clinical studies was mostly high, which was also reported by other authors (15). It was somewhat lower in older than in younger patients, and higher in better educated than in less educated ones. Similar findings have been also reported by others (25). Higher GP/FD-assigned importance to participant protection in industry-sponsored studies was associated with lower “value” assigned by patients to the general benefits of such studies. This suggests that if GPs/FDs put more emphasis on participant safety and protection, patients might have “downgraded” the general value of sponsored studies. The association between more positive GPs/FDs’ general attitude toward sponsored studies and lower patients’ agreement about the general values of such studies might appear counterintuitive. However, more positive GPs/FDs’ attitudes were at the same time associated with higher patient-assigned importance to potential benefits. Taken together, these observations suggest the possibility that a more positive general GPs/FDs’ attitude “transferred” the message that emphasized “positivity” of the potential patients’ benefits as a motivating factor, and not of the “general values” of sponsored studies.

Patients generally did not perceive industry-sponsored clinical studies as a particular health risk for the participants. The association between higher GPs/FDs’ appreciation of the general values of sponsored studies with lower patients’ agreement about the associated risks also suggests that some “transfer” of “reassuring” views might have occurred.

Overall, patients assigned moderate importance to potential personal benefits associated with participation in industry-sponsored studies – altruistic, rather than “egoistic” motives for participation in clinical research have been reported in developed (15) and developing countries (26). The fact that higher GPs/FDs’ agreement about the general values of sponsored studies and higher patients’ education were each associated with lower patient-assigned importance to personal benefits supports the view that this benevolence might arise from better education, but that it also might be “influenced” by a transfer of GPs/FDs’ views. The observations about patients’ (general) willingness to participate in sponsored clinical research are in line with such a concept: i) higher GPs/FDs’ agreement about the general values of sponsored studies was associated with higher odds of patients’ willingness; ii) older GPs/FDs’ age was also associated with higher odds of patients’ willingness, which might suggest that older, more experienced GPs/FDs were able to “transfer” more positive attitudes (during the present interview or at any prior time); iii) higher patients’ education was strongly associated with higher odds of willingness; iv) willingness was progressively higher at higher levels of patients’ appreciation of the general values of sponsored studies, and progressively lower with higher patients’ appreciation of potential risks; v) there was a quadratic relationship between patient-assigned importance to potential personal benefits and their willingness to participate: when the assigned importance was low, willingness was high (might indicate “altruistic” individuals), it then decreased toward “middle-range” importance of personal benefits, and increased at higher levels of assigned importance (might indicate “egoistic” individuals). Expectedly (15,25), willingness to participate was lower in older and in generally healthy patients.

Although the observed associations between the GPs/FDs’ and patients’ attitudes might not be dramatic, the present data indicate that even without specific interventions, in “a native sample” of GPs/FDs and their patients, some “transfer” of views might have happened in the previous contacts or during the current interaction. One study targeting community-based physicians suggested that their general views on sponsored clinical trials were generally affirmative, but with many misconceptions (27), and that these could be rectified by short online educational courses (28). In the context of the present survey, this indicates that GPs/FDs might benefit if provided more detailed and accurate information regarding all aspects of industry-sponsored and other clinical research, and that this information would be, at least to some extent, conveyed to their patients, ie, to the general public. This could contribute to forming positive attitudes toward clinical research, improve acceptance of the results of medical investigations, and contribute to successful delivery of adequate health care. The recent experience with vaccines against the SarsCov-2 virus is an illustrative example (29,30). The fact that in the present survey, patients generally assigned limited importance to advice from their GP/FD (somewhat more if uncertain about potential risks or interested in potential benefits) may seem to contradict such a possibility. However, in the present survey the question about GP/FD consultations pertained to a hypothetical situation. Investigations targeting specific patient groups and specific clinical studies indicated high patient appreciation of consultations with and advice from their GPs/FDs (23,24).

The present study suffers from several limitations. The major one is common to all surveys based on voluntary participation – selection bias (self-selection) is unavoidable, and the results are not generalizable to any particular population (31-33). In agreement, we did not intend to infer about European family doctors and their patients, but rather to test the hypothesis that their attitudes toward the topic might be related – a purpose for which non-probability (eg, volunteer, convenience) samples may be suitable (34,35). We therefore refrained from formal statistical tests to avoid their misinterpretation, and preferred to address “associations” mainly in a qualitative sense, and used confidence intervals around the association measures to communicate uncertainty about their directions. For the following several reasons we believe that the observed relationships hold within the present sample, and that data are strongly suggestive of the associations between GPs/FDs’ attitudes and attitudes of their patients in general (34,35): i) a reasonable number of GPs/FDs’ and patients’ characteristics were considered as covariates in all models; ii) proportion of missing data on variables of primary interest was reasonably low; iii) exploratory factor analysis and reasonable Cronbach alpha values indicated that the intended constructs were captured; iv) similar values of all outcome variables across regions (very low or no variability) may be viewed as correlates of similar values from different samples (suggestive of reproducibility). Next, although the data were cross-sectional, and although the doctor-patient communications were most likely (and hopefully) bidirectional (particularly in the case of better educated patients) (36), we *a priori* conceived an analysis in which GPs/FDs attitudes were “independents” and patients’ attitudes were the outcomes. We considered that, in this specific matter, and presuming their higher level (be it real or perceived) of subject matter knowledge, it was justified to “assign” the role of an educator to GPs/FDs (as it would be done in any question pertinent to an individual patient’s health) (37,38). We do not see this as a major fallacy, because the assigned “roles” of predictors and outcomes pertained only to the analytical level – we intended no inference about the possible true direction of a presumed association, just its existence. However, the survey set-up supports this approach: it is the GPs/FDs’ attitudes that were first recorded (not related to interaction with patients), and then interviews with the patients followed. Finally, we did not address communication skills (verbal, nonverbal) of the included physicians, which are known to vary considerably (39) – it could be (due to, eg, selection bias) that the observed associations resulted from the fact that only “better communicators” responded to the invitation to participate, and were able to encourage their patients to participate. Indirectly, however, the similarity of general characteristics and attitudes declared by the 201 patient-enrolling and 56 non-enrolling GPs/FDs, does not support this as a likely “decisive” element.

In conclusion, in a convenience sample of GPs/FDs and their patients from nine European countries we observed associations between GPs/FDs attitudes and attitudes of their patients regarding industry-sponsored clinical studies, which were also related to willingness to participate in such studies.
