# Supplementary material for: Is there a relationship between attitudes of general practitioners/family doctors and attitudes of their patients regarding industry-sponsored clinical investigations? A cross-sectional survey in a convenience sample of doctors and patients across nine European countries
Source: Croat Med J. 2024 Aug;65(4):313–27. doi: 10.3325/cmj.2024.65.327 (PMC11399717; doi:10.3325/cmj.2024.65.327)
Supplement: Supplementary Material [file CroatMedJ_65_s004.pdf]

Supplemental Material

Marković Zoya M et al. Is there a relationship between attitudes of general practitioners/family doctors and attitudes of their patients regarding industry-sponsored clinical investigations? A cross-sectional survey in a convenience sample of doctors and patients across 9 European countries

Contents

A. Pilot patient data: exploratory factor analysis 2
B. Missing data on attitude-related items and data imputation: GPs/FDs 2
C. Missing data on attitude-related items and data imputation: patients 5
D. Exploratory factor analysis of attitude-related items in GP/FD questionnaires 6
E. Exploratory factor analysis of patients' attitude-related items 9
F. Categorization of countries by "presence" of industry-sponsored research 9
G. Characteristics of GPs/FDs not included in the present analysis 11
H. Relationship bertween doctors' and patients' attitudes towards sponsored clinical research – model building 12
I. Relationship of GP/FDs' attitudes and of patients' attitudes with patients' willingness to participate in a potential industry-sponsored study – model building 14
J. Patients' attitudes towards industry-sponsored studies and importance that they assigned to advide by their GP/FD regarding potential participation – model building 15

A. Pilot patient data: exploratory factor analysis

The number of GPs/FDs who responded to the pilot survey was lower than expected, hence 75 patients were enrolled instead of the intended 100. Aware of this limitation, we still performed exploratory factor analysis [principal axis factoring, quatrimax rotation; IBM SPSS for Windows, version 25 (IBM Corp., Armonk, NY)]. As shown in Table S1, three factors were identified, corresponding to the three intended latent constructs: a) Importance of potential personal benefits (from trial participation); b) agreement about potential risks (of trial participation) and c) agreement about general values of industry-sponsored clinical research.

Table S1. Summary of exploratory factor analysis of items from the pilot study pertaining to patient attitudes towards industry-sponsored clinical research (N=75).

	Extraction		Factors and loadings		
Item	KMO	Commun.	Importance of personal benefits	Agreement about potential risks	Agreement about general benefits
I could get new treatments, otherwise unavailable	0.690	0.621	0.728		
I could continue using new treatments after a trial	0.699	0.447	0.640		
The study sponsor is liable for any harm that I could experience	0.650	0.901	0.922		
Industry-sponsored clinical research is unethical	0.774	0.401		0.574	
Participation in industry-sponsored clinical research could harm my health	0.706	0.647		0.787	
Industry-sponsored clinical research contributes to improvement of population health and develop.	0.706	0.355			0.551
Increasing the number of sponsored clinical research would contribute to improvement of people's health in my country	0.702	0.883			0.841
Overall	0.696	--	Total variance explained: 60.8%		

KMO – Kaiser-Meier Olkin measure of sampling adequacy; Commun. - communality

B. Missing data on attitude-related items and data imputation: GPs/FDs

Overall, 257 GPs/FDs returned the questionnaires, but 56 of them (21.8%) recruited no patients and were not included in the present analysis. As shown in Table S2, the pattern of missing data for the 201 included and 56 not included GPs/FDs was generally similar. We implemented regression-based multiple imputation (fully conditional specification) with minimum-maximum values constrained at theoretically possible values, separately for the included and not included GPs/FDs. For each subset, we generated 5 imputed datasets, but since data missingness was low, for a particular imputed item we used the imputed dataset with the smallest difference in proportion of responses vs. the observed data. Table S3 summarizes proportions of responses in imputed and observed data. We used IBM SPSS, version 25 (IBM Corp., Armonk, NY).

Table S2. Missing data on attitude-related items in GP/FD questionnaires for 201 included and 56 not included GPs/FDs (enrolled no patients).

	Included GPs/FDs (N=201)			Not included GPs/FDs (n=56)		
Item	Valid N	Missing N (%)	Role	Valid N	Missing N (%)	Role
Age	201	0	Predictor	56	0	Predictor
Sex	201	0	Predictor	56	0	Predictor
Country / region	201	0	Predictor	56	0	Predictor
Years of experience as a GP/FD	201	0	Predictor	56	0	Predictor
Specialist GP/FD	201	0	Predictor	56	0	Predictor
Dwelling (rural, urban, suburban)	201	0	Predictor	56	0	Predictor
Participates in student/resident education	201	0	Predictor	56	0	Predictor
Has been an investigator in a sponsored trial	201	0	Predictor	56	0	Predictor
Has been a subject in a sponsored trial	201	0	Predictor	56	0	Predictor
Number of registered patients	201	0	Predictor	56	0	Predictor
Average daily patient contacts	201	0	Predictor	56	0	Predictor
General attitude towards industry-sponsored clinical research (1–negative to 5 –positive)	201	0	Predictor	53	3 (5.3)	To be imputed
Sponsors' accountability for damages inflicted in sponsored clinical research is very important (1 –disagree to 5 –agree)	199	2 (1.0)	To be imputed	55	1 (1.8)	To be imputed
Confidentiality and protection of subject privacy in sponsored clinical research is very important (1 – disagree to 5 –agree)	197	4 (2.0)	To be imputed	53	3 (5.3)	To be imputed
Principles of evidence-based medicine should be promoted (1 – disagree to 5 – agree)	197	4 (2.0)	To be imputed	56	0	Predictor
Sponsored clinical research generates important evidence relevant for rational medical practice (1 –disagree to 5 – agree)	198	3 (1.5)	To be imputed	55	1 (1.8)	To be imputed
Evidence generated in sponsored clinical research reflects on my daily practice (1 –disagree to 5 – agree)	197	4 (2.0)	To be imputed	55	1 (1.8)	To be imputed
Implementation of evdence-based procedures improves patient care (1 – disagree to 5 – agree)	195	6 (3.0)	To be imputed	55	1 (1.8)	To be imputed
Evidence generated in sponsored clinical research is of a limited value for general/family medicine (1 – disagree to 5 – agree)	195	6 (3.0)	To be imputed	55	1 (1.8)	To be imputed
Adopitng evidence-based principles is a valuable intention, but implementation poses an extra load to the overloaded GP/FD (1 –disagree to 5 –agree)	196	5 (2.5)	To be imputed	55	1 (1.8)	To be imputed

Table S3. Proportions of response levels in attitude-related itmes in the observed and imputed data for the included (N=201) and not included (N=56) GPs/FDs.

		Included GPs/FDs		Not included GPs/FDs	
Item	Value	Original (%)	Imputed (%)	Original (%)	Imputed (%)
Sponsors' accountability for damages inflicted in sponsored clinical research is very important	1	66.3	65.7	60.0	58.9
	2	14.6	14.4	18.2	17.9
	3	9.5	10.0	12.7	12.5
	4	3.5	3.5	3.6	3.6
	5	6.0	6.5	5.5	7.1
Confidentiality and protection of subject privacy in sponsored clinical research is very important	1	86.8	86.1	88.7	87.5
	2	7.6	8.5	9.4	10.7
	3	2.0	2.0	1.9	1.8
	4	0	0	0	0
	5	3.6	3.5	0	0
Principles of evidence-based medicine should be promoted	1	0	0	---	---
	2	0.5	1.5		
	3	4.6	5.0		
	4	19.3	18.9		
	5	75.6	74.6		
Sponsored clinical research generates important evidence relevant for rational medical practice	1	0	0	1.8	1.8
	2	0.5	0.5	1.8	1.8
	3	8.1	8.0	10.9	12.5
	4	33.3	33.8	47.3	46.4
	5	58.1	57.7	38.2	37.5
Evidence generated in sponsored clinical research reflects on my daily practice	1	0.5	1.0	3.6	3.6
	2	1.5	1.5	1.8	3.6
	3	8.6	8.5	16.4	16.1
	4	37.1	37.3	41.8	41.1
	5	52.3	51.7	36.4	35.7
Implementation of evdence-based procedures improves patient care	1	0.5	2.5	1.8	1.8
	2	0	0	1.8	1.8
	3	3.1	3.0	7.3	7.1
	4	32.3	31.3	34.5	33.9
	5	64.1	63.2	54.5	55.4
Evidence generated in sponsored clinical research is of a limited value for general/family medicine	1	49.7	49.8	50.9	50.0
	2	17.4	16.9	23.6	23.2
	3	12.3	12.4	10.9	10.7
	4	11.3	11.4	9.1	10.7
	5	9.2	9.5	5.5	5.4
Adopitng evidence-based principles is a valuable intention, but implementation poses an extra load to the overloaded GP/FD	1	21.9	21.9	12.7	12.5
	2	16.3	16.9	18.2	17.9
	3	20.9	20.9	23.6	23.2
	4	20.9	20.9	23.6	25.0
	5	19.9	19.4	21.8	21.4

C. Missing data on attitude-related items and data imputation: patients

Table S4 summarizes information on missing data on items addressing patients' (N=995) willingness to participate in a sponsored clinical investigation, assigned relevance to the GP/FD's advice in this repsect, and attitudes related towards (sponsored) clinical investigations.

Table S4. Missing data in patients' questionnaires.

Item	Valid N	Missing N (%)	Role
Age	995	0	Predictor
Sex	995	0	Predictor
Country/region	995	0	Predictor
Dwelling	995	0	Predictor
Education	995	0	Predictor
Major current health issues	995	0	Predictor
Generally healthy/no major health issues	995	0	Predictor
Diabetes mellitus	995	0	Predictor
Gastrointestinal tract disorders	995	0	Predictor
Musculoskeletal/connective tissue disorders	995	0	Predictor
Respiratory tract disorders	995	0	Predictor
Coronary or cerebrovascular disease/incident	995	0	Predictor
Neurological disorders	995	0	Predictor
Renal and urinary tract disorders	995	0	Predictor
Mental disorders	995	0	Predictor
Malignancy	995	0	Predictor
If offered, would you participate in a sponsored trial?	995	0	Predictor
Importance of the GP advice in this respect	995	0	Predictor
Industry-sponsored clinical research contributes to population health and development (1 – disagree to 5 – agree)	949	46 (4.62)	To be imputed
Increasing the number of sponsored clinical research would contribute to improvement of people's health in your country (1 – disagree to 5 - agree)	947	48 (4.82)	Predictor
Industry-sponsored clinical research is unethical (1 – disagree to 5 – agree)	946	49 (4.92)	To be imputed
Participation in an industry-sponsored clinical investigation could harm my health (1 – disagree to 5 – agree)	944	51 (5.13)	To be imputed
How important to you is a possibility to get new and otherwise unavailable treatment by participation in a sponsored clinical research ? (1 – least important to 5 – most important)	947	48 (4.82)	To be imputed
How important to you is a possibility to continue treatment with a new drug after completion of a sponsored clinical investigation? (1 – least important to 5 – most important)	933	62 (6.23)	To be imputed
How important to you is sponsor's liability for any harm that you might experience during the trial? (1 – least important to 5 – most important)	939	56 (5.63)	To be imputed

For data imputation, we used the same methodology and logic as for data imputation to GP/FD attitude-related items. Table S5 summarizes proportions of response levels in the observed and imputed data.

Table S5. Proportions of response levels in attitude-related itmes in the observed and imputed data for the enrolled patients.

Item	Value	Original (%)	Imputed (%)
Industry-sponsored clinical research contributes to population health and development	1	2.7	2.9
	2	4.8	5.0
	3	16.4	17.4
	4	21.0	20.7
	5	55.0	54.0
Industry-sponsored clinical research is unethical	1	41.1	40.3
	2	20.8	20.6
	3	23.2	23.5
	4	9.8	9.8
	5	5.1	5.7
Participation in an industry-sponsored clinical investigation could harm my health	1	22.2	22.2
	2	16.0	16.1
	3	33.6	33.1
	4	15.4	15.7
	5	12.8	13.0
Increasing the number of sponsored clinical research would contribute to improvement of people's health in your country	1	3.6	5.3
	2	6.3	6.3
	3	18.7	18.4
	4	25.6	24.7
	5	45.8	45.2
How important to you is a possibility to get new and otherwise unavailable treatment by participation in a sponsored clinical research?	1	58.4	57.8
	2	16.4	16.4
	3	12.6	12.7
	4	4.5	4.8
	5	8.1	8.3
How important to you is a possibility to continue treatment with a new drug after completion of a sponsored clinical investigation?	1	44.5	44.0
	2	17.0	16.7
	3	20.4	21.1
	4	7.0	7.0
	5	11.1	11.2
How important to you is sponsor's liability for any harm that you might experience during the trial?	1	64.9	64.0
	2	9.7	9.5
	3	9.3	9.7
	4	5.4	5.6
	5	10.8	11.1

D. Exploratory factor analysis (EFA) of attitude-related items in GP/FD questionnaires

Considering the GPs/FDs who enrolled at least one patient (N=201), we conducted EFA (as described in Section A) on 9 items illustrating GP/FDs' attitudes towards sponsored clinical investigations/trials. In the first run, variable "general attitude towards industry-sponsored clinical research" showed low communality and did not clearly load on any of the 3 identified factors. It was therefore removed and considered as a separate predictor (5-level ordinal, 1 – negative to 5 – positive). The analysis was repeated on 8 items, and was performed for all 5 imputed datasets with repeatedly closely similar results. For the dataset used in the final

analysis: a) for each item, the Keiser-Meier Olkin measure of sampling adequacy was >0.525, and it was 0.670 overall; b) all communalities were >0.500: c) (the same) 3 components were identified, and total variance explained was 64.7%. All factor loadings were ≥0.678. The 3 identified factors were named "Agreement about general values of sponsored clinical research" (4 items, Cronbach alpha=0.706), "Importance assigned to subject protection and privacy in sponsored research" (2 items, Cronbach alpha=0.722), and "Skeptical about implementation of evidence-based principles in family medicine" (2 items, Cronbach alpha=0.634) (Table S6).

Table S6. Latent variables identified by EFA on 8 GP/FD attitude-related items for 201 included GPs/FDs.

	Agreement about general values of industry-sponsored clinical research	Importance assigned to subject protection and privacy	Skeptical about implementation of evidence-based principles
Principles of evidence-based medicine should be promoted	**0.801**		
Sponsored clinical research generates important evidence relevant for rational medical practice	**0.717**		
Evidence generated in sponsored clinical research reflects on my daily practice	**0.688**		
Implementation of evdence-based procedures improves patient care	**0.678**		
Sponsors' accountability for damages is very important		**0.872**	
Confidentiality and subject privacy in sponsored clinical research is very important		**0.876**	
Evidence generated in sponsored clinical research is of a limited value for general/family medicine			**0.792**
Adopitng evidence-based principles is valuable, but implementation poses an extra load to the overloaded GP/FD			**0.834**

The same procedure was repeated also for 56 GPs/FDs who recruited no patients, so that the included and not included GPs/FDs could be compared not only on general characteristics but

also regarding the attitudes. Again, the item "general attitude towards industry-sponsored clinical research" had to be removed, and repeated EFA on 8 items in all 5 imputed datasets yielded similar results and identified the same three latent variables as in the analysis of data on included GPs/FDs. In the selected (most similar to the observed) imputed dataset: a) for each item, KMO measure of sampling adequacy was >0.500, and it was 0.715 overall; b) all communalities were >0.476: c) the same 3 factors were identified, and total variance explained was 62.6%. All factor loadings were ≥0.491 (Table S7).

Table S7. Latent variables identified by EFA on 8 GP/FD attitude-related items for 56 not included GPs/FDs.

	Agreement about general values of industry-sponsored clinical research	Importance assigned to subject protection and privacy	Skeptical about implementation of evidence-based principles
Principles of evidence-based medicine should be promoted	**0.830**		
Sponsored clinical research generates important evidence relevant for rational medical practice	**0.712**		
Evidence generated in sponsored clinical research reflects on my daily practice	**0.658**		
Implementation of evdence-based procedures improves patient care	**0.846**		
Sponsors' accountability for damages is very important		**0.491**	
Confidentiality and subject privacy in sponsored clinical research is very important		**0.803**	
Evidence generated in sponsored clinical research is of a limited value for general/family medicine			**0.728**
Adopitng evidence-based principles is valuable, but implementation poses an extra load to the overloaded GP/FD			**0.815**

E. Exploratory factor analysis (EFA) of patients' attitude-related items

We conducted EFA on 7 patients' attitude-related items (as explained in Section A). EFA was repeated for all 5 imputed datasets, and always yielded similar results. Regarding the used dataset: a) for each item, KMO measure of sampling adequacy was >0.500, and it was 0.707 overall; b) all communalities were ≥0.543; c) 3 factors were identified, and total variance explained was 69.1%. All factor loadings were ≥0.588. (Table S8). Factors were named (i) "Agreement about general values of sponsored clinical research" (2 items, Cronbach alpha=0.650), (ii) "Agreement about potential risks" (2 items, Cronbach alpha=0.646) , and (iii) "Importance assigned to potential personal benefits" (3 items, Cronbach alpha=0.751).

Table S8. Latent variables identified by EFA on 7 patients' attitude-related items.

	Agreement about general values	Agreement about potential risks	Importance of personal benefits
Industry-sponsored clinical research contributes to population health and development	**0.831**		
Increasing the number of sponsored clinical research would contribute to improvement of people's health in your country	**0.830**		
Industry-sponsored clinical research is unethical		**0.588**	
Participation in an industry-sponsored clinical investigation could harm my health		**0.917**	
How important to you is a possibility to get new and otherwise unavailable treatment by participation in a sponsored clinical research?			**0.781**
How important to you is a possibility to continue treatment with a new drug after completion of a sponsored clinical investigation?			**0.846**
How important to you is sponsor's liability for any harm that you might experience during the trial?			**0.812**

F. Categorization of countries by "presence" of industry-sponsored research

To additionally justify classification of countries into "regions" based on the "presence" of industry-sponsored clinical research we accessed *ClinicalTrials.gov* (Home | ClinicalTrials.gov) (August 14, 2023) and analyzed the preceding 5-year period (August 1, 2018 to July 31, 2023). During this period, there were 166066 clinical investigations registered, 123678 (74.5%) of which were declared as "interventional", and there were 34193 interventional clinical investigations sponsored by the industry. The absolute number of industry-sponsored interventional clinical studies was higher in larger countries (e.g., Poland, Portugal, Spain,

Turkey) than in smaller ones (Croatia, Bosnia & Herzegovina, Serbia, Montenegro, North Macedonia) (Table S9).

Table S9. The number of industry-sponsored interventional clinical studies registered at *ClinicalTrials.gov* (Clinical Trials register) between August 1, 2018 and July 31, 2023, for which the respective country is listed as a "site" or one of the "sites" included. Shown is the absolute number of studies (N), and number per 100000 population. Shown is also the percentage of the total number of studies that list the respective countries as "sites".

	N	per 100000 population	Percent of studies in which included
Croatia	229	5.9	0.7
Bosnia & Herzegovina	52	1.6	0.2
Serbia	312	4.3	0.9
Montenegro	1	0.2	0.00
North Macedonia	26	1.2	0.08
Poland	2179	5.7	6.4
Portugal	582	5.6	1.7
Spain	3585	7.5	10.5
Turkey	909	1.1	2.7

The commonly used indicator – number of studies/100000 population – suggested that Croatia and Serbia were, in this respect, similar to Poland, Portugal and Spain, and with a higher number of trials/100000 inhabitants than Turkey. However, both these indicators provide only a part of information and could be, each on its own, deceptive regarding the "presence" of industry-sponsored studies in a country. It is intuitive that larger countries can "offer" larger number of sites and (experienced) investigators for patient recruitment, and a larger pool of potential study subjects, which attracts industry sponsors. A larger absolute number of studies means that they are "available" to a larger number of investigators and study participants. On the other hand, if the question is reformulated to read – what number of industry-sponsored studies is "available" to a defined pool of people per country? – then, number of trials/100 000 inhabitants seems a better indicator. However, what is not recorded in the *ClinicalTrials.gov* statistics is the number of centers/sites in a particular country that participate in a particular study, and the number of enrolled patients: a country will be listed as one of the sites even if only one site-center and only one patient is included, just as in the case of a country with e.g., 20 participating sites and a large number of included patients. We therefore attempted to supplement the information about the "presence" of industry-sponsored clinical investigations in the countries participating in the present survey by an additional indicator that uses the same denominator for each country – i.e., the total number of industry-sponsored interventional studies registered

during the observed period, which is 34193. By this indicator, Poland, Portugal, Spain and Turkey differred from other 5 countries listed in Table S9. Overall, considering all three types of data in Table S9, we considered it justified to conclude: as compared to Croatia, "presence" of industry-sponsored research could be judged as (i) somewhat lower in Serbia, (ii) considerably lower in Bosnia & Herzegovina, Montenegro and North Macedonia, (iii) somewhat higher in Turkey and (iv) clearly higher in Poland, Portugal and Spain.

G. Characteristics of GPs/FDs not included in the present analysis

Table S10. Characteristics of GPs/FDs not included in the present analysis.

	All	Croatia	BH, Serb, MN, North Maced.	Portugal & Spain
N	56	36	6	14
Female	43 (76.8)	28 (77.8)	6	9 (64.3)
Age	46±12 (25-67)	52±9 (25-67)	48±8 (38-59)	30±4 (26-38)
Years of experience as GP/FD	17±12 (1-43)	23±11 (1-43)	17±10 (6-32)	3±2 (2-11)
Dwelling				
Urban	30 (53.6)	19 (52.8)	5	6 (42.9)
Suburban/rural	26 (46.4)	17 (47.2)	1	8 (57.1)
Specialist	35 (62.5)	22 (61.1)	6	7 (50.0)
Educates students/resident	38 (67.9)	23 (63.9)	4	11 (78.6)
Has been an investigator in a clinical research	20 (35.7)	14 (38.9)	3	3 (21.4)
Has been a study subject in a clinical research	3 (5.4)	2 (5.6)	1	0
Registered patients (x 1000)	1.80 (1.42-1.97)	1.79 (1.31-2.05)	1.87 (1.69-2,01)	1.79 (1.55-1.83)
Average daily patient contacts	55 (30-80)	80 (54-85)	40 (39-55)	18 (15-24)
Attitude towards sponsored studies				
1 - negative	21 (37.5)	11 (30.6)	0	10 (71.4)
2	16 (28.6)	12 (33.3)	0	4 (28.6)
3	11 (19.6)	7 (19.4)	4	0
4	5 (8.9)	4 (11.1)	1	0
5 - positive	3 (5.4)	2 (5.6)	1	0
Agreement about general values of industry-sponsored research*[1]	17 (16-19)	18 (16-29)	16 (15-19)	17 (16-18)
Importance assigned to subject protection/privacy*[2]	2 (2-4)	2 (2-4)	2.5 (2-5)	2 (2-3)
Skeptical about implementation of evidence-based procedures*[3]	5 (4-6)	5 (3-7)	5.5 (2-8.5)	5 (5-6)
Own practice is evidence-based?				
<5%	1 (1.8)	1 (2.8)	0	0
Around 10-30%	3 (5.4)	3 (8.4)	0	0
Around 40-50%	2 (3.6)	2 (5.6)	0	0
Around 60-70%	24 (42.8)	11 (30.5)	4 (66.7)	9 (64.3)
Around 80%	20 (35.7)	13 (36.1)	2 (33.3)	5 (35.7)
90-100%	5 (8.9)	5 (13.9)	0	0
Did not answer	1 (1.8)	1 (2.8)	0	0

Data are mean±standard deviation (range), median (quartiles) or count (percent)

*Varibales identified in exploratory factor analysis. Values are sums of individual items.

[1]Minimum possible score 4, maximum 20, mid-point 12; [2,3] Minimum possible score 2, maximum 10, mid-point 6. Higher values = higher level or agreement/assigned importance/skepticism

H. Relationship between doctors' and patients' attitudes towards sponsored clinical research – model-bulding

GP/FDs' general attitude towards industry-sponsored clinical research and the three attitude-related variables indetified through EFA were mildly correlated (Figure S1): i) progressively less subjects expressed positive *general attitudes towards industry-sponsored clinical research* and higher *importance to subject protection/privacy in such research,* hence the two variables were positively correlated (tau=0.1723, P<0.001); ii) progressively more subjects expressed higher *agreement about the general values of industry-sponsored research*, hence this variable negatively correlated with the previous two (tau=-0.2107, P<0.001; and tau= -0.162, P<0.001, respectively); iii) the proportion of subjects expressing higher level of *skepticism about implementation of evidence-based procedures in daily family practice* also displayed a declining tendency, hence this variable negatively correlated with *agreement about general values of industry-sponsored research*.

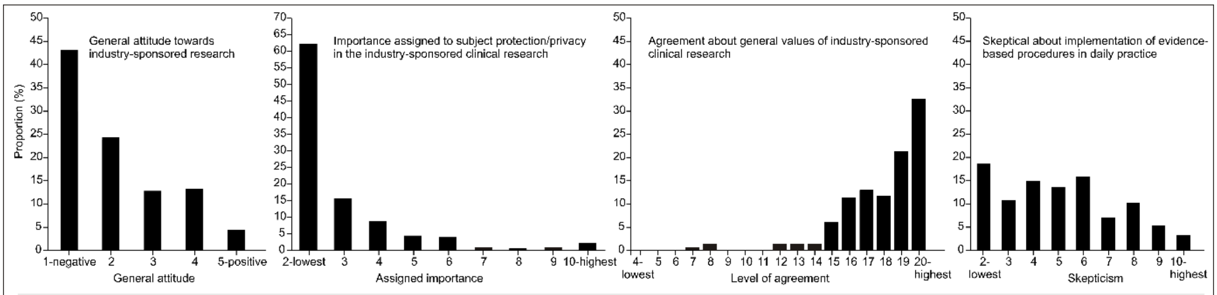

Variable 1	Variable 2	Kendall tau	P	-.8 -.6 -.4 -.2 0 .2 .4 .6 .8
Importance of subject protection	General attitude towards sponsored research	0.1723	<.0001	
General values of sponsored research	General attitude towards sponsored research	-0.2107	<.0001	
General values of sponsored research	Importance of subject protection	-0.1620	<.0001	
Skeptical about implementation in practice	General attitude towards sponsored research	0.0089	0.6879	
Skeptical about implementation in practice	Improtance of subject protection	0.0336	0.1353	
Skeptical about implementation in practice	General values of sponsored research	-0.2251	<.0001	

Figure S1. Relationship between GP/FDs' variables representing their attitudes related to industry-sponsored clinical research. Shown are distributions across the recorded values and univariate non-parametric correlations (Kendall's tau).

Similarly, patients' attitude-related variables were mildly correlated (Figure S2): i) negative correlation between *agreement about general values of sponsored research* and *agreement about potential risks of such research* (tau= -0.3402, P<0.001) and *importance assigned to potential personal benefits* (tau= -0.2252); ii) positive correlation between *agreement about potential risks* and *importance of potential personal benefits* (tau= 0.1717, P<0.001).

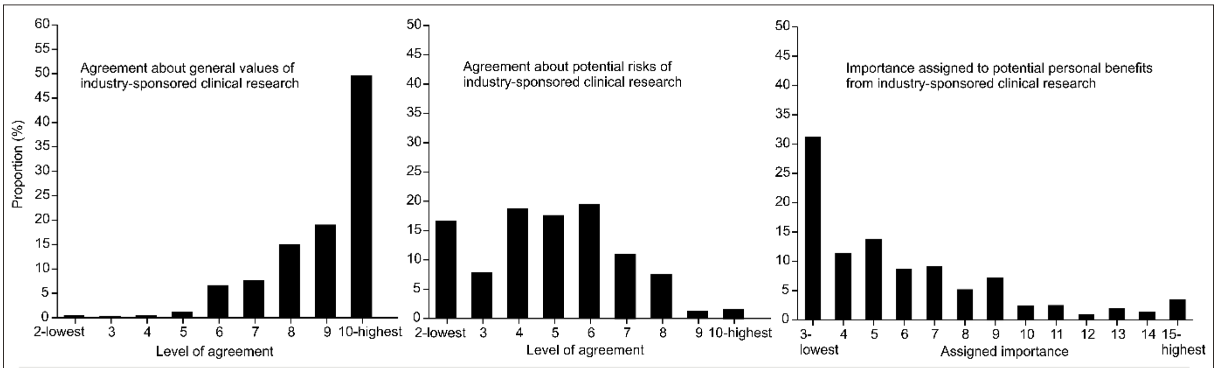

Variable 1	Variable 2	Kendall tau	P	- ,8 - ,6 - ,4 - ,2 0 ,2 ,4 ,6 ,8
Agreement about potential risks	Agreement about general values	-0.3403	<.0001	
Importance of personal benefit	Agreement about general values	-0.2252	<.0001	
Importance of personal benefit	Agreement about potential risks	0.1717	<.0001	

Figure S2. Relationship between patient variables representing their attitudes related to industry-sponsored clinical research. Shown are distributions across the recorded values and univariate non-parametric correlations (Kendall's tau).

To evaluate the relationships of primary interest (between GP/FDs' and patients attitudes), we generated a directed acyclic graph (DAG) to illustrate the concept of data analysis (Figure S3): i) although the study was cross-sectional, we assumed that if an "influence" existed (exerted either during the past doctor-patient contacts, or during the current inverivew), it was one directed from GPs/FDs towards patients (depicted by a thick arrow extending from GP/FDs' to patients' attitudes); ii) since both the GP/FDs' and patients' attitude-related variables were mutually related, an association between any of the GP/FDs'variables (predictors of interest) and any of the patients' variables (outcomes) could be to some extent mediated through any other GP/FDs' or patients' attitude variables. To avoid "blockage" of the mediated paths between individual predictors and individual outcomes of interest, each outcome was analyzed separately, and multivariate models always included only one of the predictors while other GP/FDs' and patients' attitude variables were **not in the model.**

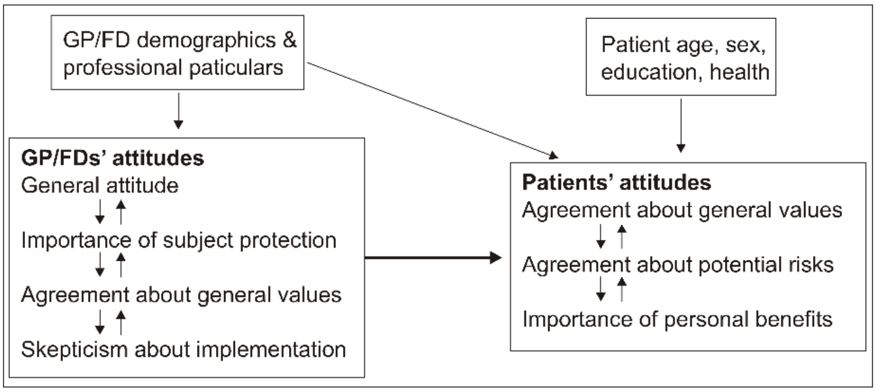

Figure S3. The conceptualized relationship between GP/FD-level and patient-level predictors and the outcomes of interest (patients' attitudes). See text for details.

Three types of models were fited: i) univariate models, one for each of the GP/FDs' attitude variable vs. each of the outcome variables; ii) multivariate models, one for each of the outcomes, with covariates only – GP/FDs' and patients' basic characteristics could have confounded the relationship between their attitudes (Figure S3). Models with covariates only estimated their "total" associations with the outcomes; iii) multivariate models, one for each of the GP/FDs' attitude variables and each outcome, but including covariates. Considering the distribution of subjects across the levels of PD/FDs' attitude-related variables (Figure S1), they were collapsed to two or three levels:

- General attitude towards sponsored research: 3-5 (more positive) vs. 1-2 (negative)
- Importance of subject protection: 4-10 (moderate-to-high) vs. 2-3 (low)
- General values of sponsored research: 17-20 (high) vs. <17 (low-to moderate)
- Skepticism about implementation in daily practice: 2-4 (low), 5-7 (intermediate), 8-10 (high)

I. Relationship of GP/FDs' attitudes and of patients' attitudes with patients' willingness to participate in a pontential industry-sponsored study – model-bulding

A DAG in Figure S4 depicts the concept of data analysis: i) the relationships of primary interest are depicted by thick arrows whose direction implies possible "influence": GP/FDs' attitudes → outcome, either "through" patients' attitudes (patients' attitudes → outcome) or "directly"; ii) GP/FDs' and patients' basic characteristics are possible confounders of the relationships of primary interest; iii) relationship of each individual GP/FDs' attitude variable with the outcome could be mediated by one or more other GP/FDs' attitude variables and any of the patients' attitude variables; iv) relationship of each individual patients' attitude variable with the outcome could be mediated by one or more other patients' attitude variables.

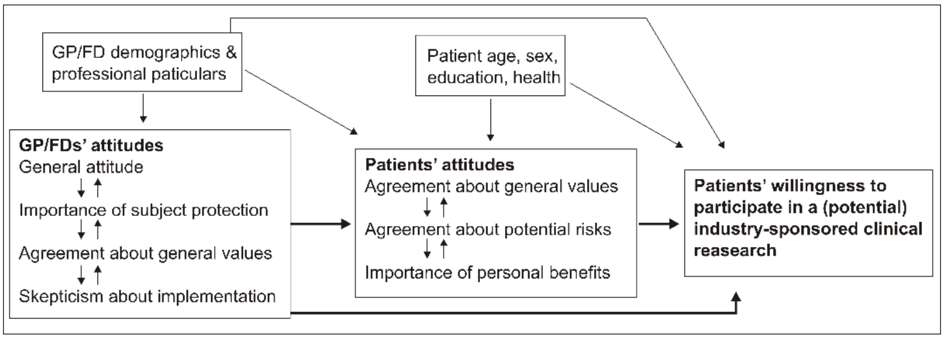

Figure S4. The conceptualized relationship between the GP/FD- and patient-level predictors and the outcome (patients' willingness to participate in a hypothetical sponsored study) (see text for details).

To avoid bias arising from adjustment for mediators, several models were fitted: i) univariate association between GP/FDs' attitudes and the outcome – one model for each attitude variable; ii) univariate association between patients' attitudes and the outcome – one model for each attitude variable; iii) a multivariate model containing only covariates (basic GP/FDs' and patients' characteristics) (assesses their "total" association with the outcome); iv) multivariate models, one for each GP/FDs' attitude variable and one for each patients' attitude variable with basic GP/FDs' and patients' characteristics as covariates.

J. Patient's attitudes towards industry-sponsored studies and importance they assigned to advice by their GP/FD regarding potential participation – model-bulding

A DAG in Figure S5 depicts the concept of data analysis: i) in the present sample, without a specific intervention (e.g., a workshop intended to inform GP/FDs about key particulars of sponsored clinical research, followed by educational sessions by GP/FDs for their patients), it was highly unlikely that GP/FDs' attitudes about industry-sponsored clinical studies would be relevant for the level or importance assigned by their patients to a GP/FDs' advice about participation in such studies. This is indicated **by the lack** of a box "GP/FD attitudes" in Figure S5; ii) we reasoned that the level of assigned importance was primarily affected by the patients' general particulars, i.e., demographics, education, general health, either "directly" or through paths that involve mediators, like patients' attitudes towards the sponsored studies and/or willingness to participate in such studies; iii) we also reasoned that the level of importance assigned by the patients to the advice by their GP/FD was affected by the patients' general appreciation of their GPs/FDs (i.e., general level of trust), which could be driven by general (demographic, professional) GPs/FDs' characteristics.

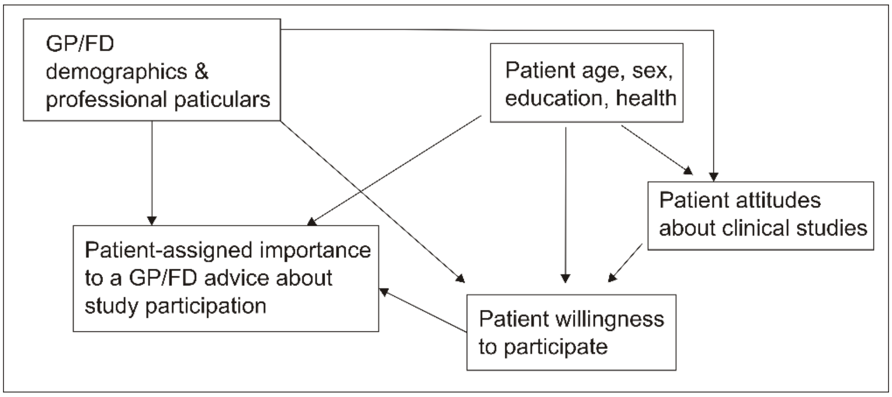

Figure S5. The conceptualized relationship between GP/FD and patient-level predictors and the outcome – patient-assigned importance to a GP/FDs' advice about study participation. See text for details.

Since by far most of the patients assigned very low or low importance (on a scale from 1-lowest to 5-highest, 78.9% patients assigned 1 or 2 points, only 8.9% assigned 3 points, and only 21.1% assigned cumulativey 3-5 points – see Table 2 in the main text) to consultations/advice by their GP/FD regarding participation in a (potential, hypothetical) industry-sponsored clinical study, the outcome was dichotomized as "moderate to high importance" (3-5 points) vs. "very low or low importance" (1-2 points). To avoid bias arising from adjustment for mediators, we gradually fitted several logit models to the outcome: i) a separate univariate model for each of the three patients' attitude variables (agreement about general values, agreement about potential risks, and importance of potential personal benefits), and for patients' willingness to participate in such a study; ii) a multivariate model containing only covariates (basic GP/FDs' and patients' characteristics) – assessed their "total" associations with the outcome; and iii) multivariate models – one for each of the patients' attitude variables and willingness to participate with all covariates.
